# Supplementary material for: Intrinsic and Extrinsic Transcriptional Profiles That Affect the Clinical Response to PD-1 Inhibitors in Patients with Non–Small Cell Lung Cancer
Source: Cancers (Basel). 2022 Dec 29;15(1):197. doi: 10.3390/cancers15010197 (PMC9818269; doi:10.3390/cancers15010197)
Supplement: Supplementary file 1 [file cancers-15-00197-s001.zip › cancers-2051171-supplementary-done.pdf]

cancers

MDPI

Supplementals

Intrinsic and Extrinsic Transcriptional Profiles that Affect The Clinical Response to PD-1 Inhibitors in Patients with Non–Small Cell Lung Cancer

Hye Eun Byeon [1], Seokjin Haam [2], Jae Ho Han [3], Hyun Woo Lee [4] and Young Wha Koh [3]

Table S1. Demographic and clinical characteristics of patients.

Variable	Adenocarcinoma (*n* = 31) (%)	Squamous cell carcinoma (*n* = 26) (%)
Age, median (range) (years)	62 (40–84)	71 (43–85)
Male sex	22 (71%)	23 (88.5%)
Smoking history	15 (68.2%)	23 (92%)
TNM stage		
III	4 (12.9%)	9 (34.6%)
IV	27 (87.1%)	17 (65.4%)
Specimen type		
Biopsy	21 (67.7%)	19 (73.1%)
Operation	10 (32.3%)	7 (26.9%)
PD-1 inhibitor		
Pembrolizumab	17 (54.8%)	16 (61.5%)
Nivolumab	14 (45.2%)	10 (38.5%)
Genetic alteration status		
EGFR-mutated	12 (40%)	0 (0%)
ALK-rearranged	0 (0%)	0 (0%)
PD-L1 expression		
>50%	22 (71%)	18 (69.2%)
Response to PD-1 blockade		
Responder	7 (22.6%)	6 (23.1%)
Non-responder	24 (77.4%)	20 (76.9%)

Smoking history was collected for 22 patients for adenocarcinoma and 25 patients for squamous cell carcinoma; EGFR test was performed for 30 patients for adenocarcinoma and 26 patients for squamous cell carcinoma.

Table S2. The gene list of 10 pathways.

Activating Invasion and Metastasis (*n* =149)
ACTR3, AKT1, AMOTL2, AR, AURKA, AXL, BIRC5, CASP3, CAV1, CCL2, CCL21, CCR7, CD274, CD44, CD47, CDC20, CDH1, CDH11, CDH5, CEACAM1, COL4A1, COL4A2, COL6A1, COL6A2, COL6A3, CTNNA1, CTNNB1, CTNND1, CXCR4, DCN, DNMT1, DOCK1, EGF, EGFR, EPB41L3, EPCAM, ERBB2, ESR1, ESRP1, EZH2, FBLIM1, FBLN2, FBN1, FGFR1, FLT4, FMOD, FOXA1, FOXM1, FRMD6, FSTL1, GFPT2, GREM1, GRHL2, GSK3B, HDAC1, HGF, HIF1A, IGF1R, IL6, ITGA1, ITGA11, ITGA5, ITGA8, ITGAL, ITGAM, ITGAV, ITGAX, ITGB1, ITGB2, ITGB3, ITGB5, ITGB6, ITGB8, JAK2, JUN, JUP, KDM1A, KLK2, KRAS, LAMA2, LAMA4, LAMB1, LAMB3, LAMC1, LAMC2, LATS1, LCN2, LOX, LTBP1, MAPK1, MAPK3, MET, MMP9, MTOR, MUC1, MYC, MYL9, NANOG, NFKB1, PAK2, PARD3, PARVA, PDPK1, PGF, PIK3CA, POU5F1, PTEN, PTGS2, RAC3, RAF1, ROCK1, ROCK2, SAV1, SDC1, SERPINE1, SHH, SLIT2,

SMAD7, SNAI1, SNAI2, SOX2, SRC, STAT3, STK3, STK4, TAZ, TEAD1, TEAD2, TGFB1, TGFB2, TGFB3, TGM2, THBS1, THBS2, TJP1, TLR4, TNF, TNS1, TOP2A, TP53, TP73, TPSAB1/B2, VEGFA, VWF, WNT5A, WWC1, WWTR1, YAP1, ZEB1
**Avoiding immune Destruction (n =122)**
ADORA2A, ADORA2B, ARG1, B2M, B4GALNT1, BCAP31, BLK, CARD11, CD163, CD19, CD209, CD22, CD244, CD247, CD27, CD274, CD276, CD28, CD38, CD3D, CD3E, CD3G, CD4, CD40, CD40LG, CD44, CD6, CD80, CD84, CD86, CD8A, CD8B, CDK4, CEACAM1, CTAG1A/B, CTLA4, CTSW, ENTPD1, ENTPD2, EOMES, FCRL2, FOLH1, FOS, GNLY, GPNMB, GRAP2, GZMA, GZMB, GZMH, GZMK, HAVCR2, HIF1A, HLA-A, HLA-B, HLA-C, HLA-DQA1, HLA-DRB1, HLA-E, ICOS, IDO1, IDO2, IFNG, IL10, ITK, JUN, KIR2DL3, KIR2DL4, KIR2DS4, KIR3DL1, KIR3DL2, KLK3, KLRB1, KLRD1, KLRG1, KLRK1, LAG3, LAT, LCK, MAGEC1, MAGEC2, MLANA, MS4A1, MS4A2, MS4A4A, MSLN, MST1R, MUC16, NCK1, NCR1, NFATC2, NKG7, NOS2, NT5E, PAK2, PDCD1, PDCD1LG2, PRF1, PSMB10, PSMB7, PSMB9, PTGS2, PTPN6, PTPRC, SELL, SPIB, TAP1, TAP2, TAPBP, TBX21, TCF7, TIGIT, TNF, TNFRSF18, TNFRSF4, TNFRSF9, TOX, TOX2, TOX3, TOX4, TRAT1, VAV1, ZAP70
**Deregulating Cellular Energetics (n=105)**
ABCC1, ACOT12, ACSL3, ADH1A, AKT1S1, ALDH1A3, ALDOA, ATF2, ATG101, ATG2B, ATG4B, ATOX1, ATP7A, AURKA, BHLHE40, BUB1, CACYBP, CCNF, CCS, CDC25A, CHMP2A, COX5B, CPT2, CREB1, CXCR4, CYBB, CYP4A11/22, EGLN3, EHHADH, EIF2AK3, EIF4EBP1, EIF4G1, ELOVL5, ERN1, ERO1A, GABARAP, GABPA, GLUD1, GLUD2, GLUL, GMPS, GPT, GPX1, GSR, GSTM4, HK2, HMOX1, IDH1, IDH2, ITGB2, KEAP1, LAMTOR1, LAMTOR2, LAMTOR4, LAMTOR5, LDHA, MAFG, MAPK14, MCM2, MCM4, MLST8, MTHFD2, MTOR, NCF1, NCF2, NCOA2, NCOR1, NDUFA2, NDUFB1, NFE2L2, NUDT2, P4HB, PDK1, PFKFB3, PFKP, PGK1, PIK3R4, PKM, PLK1, PRDX1, PRDX2, PRDX6, PRKACA, RB1CC1, RICTOR, RIMKLB, RPTOR, RRAGC, RRM2, SLC1A5, SLC2A1, SLC7A5, SOD1, SORD, SQLE, SQSTM1, SREBF1, STIP1, TPI1, TUBG1, TXN, TXN2, TXNRD2, UCHL5, XBP1
**Enabling Replicative Immortality (n=48)**
ABCG2, ATM, BCL2, CCNA2, CCNE1, CCNE2, CD34, CD44, CDK2, CDK4, CDK6, CDKN1A, CDKN2A, DKC1, DNA2, E2F1, E2F2, EPAS1, EZH2, FEN1, HMGA1, IGF1R, IL6, ITPR1, ITPR3, KRAS, LIG1, MAPK14, MDM2, MYC, NANOG, NFKB1, PCNA, POLE, POU5F1, PRIM1, RB1, RFC2, RFC3, RFC4, RPA3, RUVBL1, SOX2, STAT3, TERF2IP, TERT, TP53, WRAP53
**Evading Growth Suppressors (n=81)**
AURKA, AURKB, BRIP1, BUB1, BUB1B, CCNA2, CCNB1, CCNB2, CCND1, CCND2, CCNE1, CCNE2, CCNF, CCNK, CCNT1, CDC20, CDC25A, CDC25C, CDCA5, CDCA8, CDK1, CDK2, CDK4, CDK6, CDKN1A, CDKN2A, CENPA, CLSPN, COP1, DCBLD2, DKC1, DNA2, E2F1, E2F2, EXO1, FEN1, FOXM1, GTSE1, HJURP, IKZF1, KIF20A, KIF23, KIF2C, LIG1, LIN9, MCM2, MCM4, MDM2, MKI67, MYBL2, NCAPG, NEK2, NUF2, PCNA, PLK1, POLE, PRIM1, RAD51, RB1, RBL1, RBX1, RFC2, RFC3, RFC4, RPA3, RRM2, RUVBL1, SFN, SKA1, SKP2, SMC3, STAG2, TFDP1, TOP2A, TP53, TPX2, TTK, TUBG1, UBE2C, WEE1, WRAP53
**Genome Instability & Mutation (n=111)**
AGO2, AGO4, ARID1A, ARID1B, ARID2, ARID4A, ARID4B, ASH1L, ASXL2, ATF2, ATF4, ATF7IP, ATM, ATR, ATRX, ATXN7, BAX, BCL2A1, BCL2L1, BIRC3, BLM, BRCA1, BRCA2, BRIP1, BRMS1, CBX2, CCNK, CCNT1, CD14, CD40, CD86, CDK12, CHEK1, CHEK2, CLOCK, CLSPN, COP1, CREBBP, CSF1R, DICER1, DNA2, DTL, EME1, EP300, EPC1, EXO1, EZH2, FANCA, FANCD2, FANCI, FEN1, GTSE1, H2AX, HDAC1, JMJD1C, KAT2B, KAT6A, KAT6B, KDM1A, KDM3B, KDM5A, KDM6A, KLF5, KMT2A, KMT2D, KPNA2, LIG1, MDM2, MLH1, MSH2, MYC, NCOR1, NEIL3, NSD1,

PARP1, PARP2, PARP3, PBRM1, PCLAF, PCNA, PMAIP1, POLE, RAD51, RAD51AP1, RAD54B, RAD54L, RBBP5, RNF111, RPA3, RUNX1, RUVBL1, SERPINB5, SETD2, SF3B1, SIN3A, SOD1, SPINT1, SSRP1, SUPT16H, TET2, TIMELESS, TMPRSS2, TP53, TP73, TRAF1, TRRAP, UBE2T, VCP, XRCC2, ZEB1, ZMYM2
**Inducing Angiogenesis (n=69)**
ABI1, ADM, ALDOA, ANGPT2, CA9, COL4A1, COL4A2, COL6A1, COL6A2, COL6A3, CRK, EDN1, EGLN3, EP300, FLT1, FLT4, GRB7, HIF1A, HK2, HMOX1, HRAS, HSPB1, IGFBP3, KDR, LDHA, LTBR, MAPK13, MAPK14, NCK1, NOS2, NOS3, NRAS, P4HA2, PDGFB, PDGFRA, PDGFRB, PDK1, PFKFB3, PFKP, PGK1, PIK3CA, PIK3CB, PIK3R1, PIK3R2, PKM, PLA2G4A, PLCG2, PRKCB, RASA1, RBX1, SERPINE1, SLC2A1, SOS1, SPHK2, SRC, STAT1, STAT3, TEK, TGFB3, THBS1, THBS2, TPI1, VEGFA, VEGFB, VEGFC, VEGFD, WASF2, NRP1, HBEGF
**Resisting Cell Death (n=24)**
BAD, BAX, BCL2, BCL2A1, BCL2L1, CASP3, CD27, FAS, FASLG, GZMB, LMNB1, MAPK8, PMAIP1, TNF, TNFRSF17, TNFRSF18, TNFRSF4, TNFRSF9, TNFSF11, TNFSF13B, TNFSF14, TNFSF8, TP53, TNFRSF1B
**Sustaining Proliferative Signaling (n=225)**
ABCC4, ABL1, ACSL3, ACTG2, AGO2, AGO4, AIMP2, AKT1, AKT1S1, AKT2, AKT3, ALDH1A3, ALK, APC, AR, ARID1A, ATF2, ATF4, B4GALT1, BHLHE40, BMPR1A, BMPR2, BRAF, BRCA1, BTK, BYSL, CACYBP, CALML3, CCNA2, CCNE1, CCNT1, CDCP1, CDK1, CDK4, CDK6, CHD8, CREB1, CRK, CSF2RA, CSF2RB, CTNNB1, CXXC5, DCN, DLL3, DUSP6, EGF, EGFR, EIF4EBP1, ELOVL5, EP300, ERBB2, ERBB3, ESR1, ESR2, ESRP1, ESRP2, FAM83A, FASLG, FBLN2, FBN1, FGF7, FGFBP1, FGFR1, FGFR2, FGFR3, FLT3, FMOD, FOXA1, FOXP1, FZD1, FZD4, FZD7, FZD8, GALNT3, GNAI3, GRB7, GREM1, GSK3B, GSR, HDAC1, HGF, HK2, HRAS, HSPB1, IL11, IL2RA, IL2RB, IL2RG, IL3RA, IL6, INHBA, IQGAP1, ITGAV, ITGB1, ITGB3, JAG1, JAK1, JAK2, JAK3, JUN, KAT2B, KDM1A, KIT, KLK2, KLK3, KLK4, KPNA2, KRAS, KRT16, KRT17, LAMA2, LAMA4, LAMB1, LAMB3, LAMC1, LAMC2, LAMTOR2, LDHA, LOX, LRRC32, LTBP1, MALL, MAML2, MAP2K1, MAP2K2, MAP3K14, MAP3K7, MAP4K1, MAPK1, MAPK13, MAPK14, MAPK3, MAPK8, MCM4<br>MET, MGA, MYB, MYC, NBL1, NCOA2, NCOR1, NF1, NKX3-1, NME1, NOS3, NOTCH3, NRAS, P4HB, PA2G4, PAK2, PDGFB, PDGFRA, PDGFRB, PDLIM5, PHB, PIK3CA, PIK3CB, PIK3CD, PIK3R1, PIK3R2, PIK3R3, PIK3R4, PIK3R5, PLCB3, PLEK2, PLK1, PPAN, PRKACA, PRKCA, PSMB10, PSMB7, PSMB9, PTCH1, PTCH2, PTEN, PTGS2, RAC3, RAF1, RALB, RASA1, RB1, RBBP5, RBL1, RBX1, RET, RUNX1, RUVBL1, SH3PXD2A, SHH, SKIL, SKP2, SMAD7, SMC3, SMO, SMS, SORD, SOS1, SOX7, SPDEF, SPINT1, SPINT2, SRC, SRM, STAG2, STAT3, TBRG4, TCF7, TCOF1, TEK, TERT, TFDP1, TGFB1, TGFB2, TGFB3, TGFBR2, THBS1, TMPRSS2, TNS4, TPD52, TRRAP, TSPAN1, UGDH, VCP, VWF, WNT5A
**Tumor-promoting Inflammation (n=188)**
ACKR2, ACKR3, ADAMDEC1, B2M, BCL2A1, BCL2L1, BIRC3, BTK, C3AR1, CARD11, CCL13, CCL19, CCL2, CCL21, CCL4, CCL5, CCR1, CCR2, CCR4, CCR5, CCR7, CD14, CD300A, CD33, CD37, CD40, CD40LG, CD44, CD47, CD53, CD68, CEACAM1, CEACAM3, CFLAR, CGAS, CHUK, CLEC4A, CMKLR1, CRK, CSF1R, CSF2RA, CSF2RB, CSF3R, CXCL10, CXCL11, CXCL9, CXCR2, CXCR4, CXCR6, CYBB, CYFIP1, DAPP1, DOCK2, EIF4G1, F13A1, FCAR, FCER1G, FCGR3A/B, FPR1, GNAI3, GSTM4, HAVCR2, HCK, HLA-A, HLA-B, HLA-C, HLA-DQA1, HLA-DRB1, HLA-E, HUWE1, ICAM1, IFNA1, IFNAR1, IFNAR2, IFNG, IFNGR2, IKBKG, IL10, IL10RA, IL11, IL12B, IL12RB1, IL15, IL15RA, IL21R, IL22RA2, IL27RA, IL2RA, IL2RB, IL2RG, IL3RA, IL4R, IL6, IL6ST, IL7, IL7R, IL9R, IRF1, IRF3, IRF4, IRF7, IRF8, ITGAL, ITGAM, ITGAV, ITGAX, ITGB1, ITGB2, ITGB3, ITK, JAK1, JAK2, JAK3, KPNA2, LAIR1, LAPTM5, LAT,

LCK, LCN2, LILRB2, LTA, LTB, LTBR, LY96, LYN, MAP3K14, MAP3K7, MAPK3, MCL1, MKNK1, MNDA, MX1, MYD88, NFAM1, NFKB1, NFKB2, NOS2, OAS1, OAS2, OAS3, OSM, OSMR, PA2G4, PARP1, PKM, PLAU, PLCG2, PRDM1, PRKCB, PSMB10, PSMB9, PTGER1, PTGER2, PTGER3, PTGER4, PTGS2, PTPN6, RAF1, REL, RELB, S100A12, SDC1, SELL, SIGLEC5, SIGLEC9, SIRPA, SLPI, SOCS1, SOCS3, SQSTM1, STAT1, STAT3, STAT4, STING1, SYK, TLR3, TLR4, TNF, TNFAIP3, TNFSF11, TNFSF13B, TNFSF14, TRAF1, TRAF6, TYROBP, VCP, XCL1/2, ZAP70

Table S3. 100 genes involved in response to PD-1 inhibitors.

A. Non-small cell carcinoma.

	**ANOVA value**	**Fold change**	**Pathway**
IL15RA	22.655556	1.89	Extrinsic
CCR1	22.171206	2.25	Extrinsic
CCL2	21.985962	2.9	Extrinsic
CYBB	20.290789	2.52	Extrinsic
FCER1G	18.919663	2.43	Extrinsic
PDCD1LG2	15.977685	2.55	Extrinsic
CD274	15.707638	2.03	Extrinsic
FCGR3A/B	14.036684	2.2	Extrinsic
CCL13	13.920592	3.38	Extrinsic
LAG3	13.748618	2.98	Extrinsic
NKG7	13.299997	3.32	Extrinsic
CXCL10	13.083803	4.72	Extrinsic
GREM1	12.973208	3.04	Intrinsic
TAP1	12.686163	2.05	Extrinsic
CD68	12.549599	2.12	Extrinsic
CD14	12.457989	2.18	Extrinsic
LAPTM5	11.800395	1.95	Extrinsic
HAVCR2	11.799847	1.82	Extrinsic
AURKA	11.677235	1.62	Intrinsic
GZMH	11.658875	2.5	Extrinsic
CGAS	11.626901	1.62	Extrinsic
GZMB	11.568193	2.92	Extrinsic
STAT1	11.537315	1.77	Extrinsic
CMKLR1	11.326467	2.11	Extrinsic
B2M	10.675469	1.73	Extrinsic
IL2RA	10.48627	1.94	Extrinsic
AXL	10.463373	1.73	Intrinsic
RRM2	10.180753	1.77	Intrinsic
PRF1	10.10217	2.41	Extrinsic
CDC25A	10.009255	1.56	Intrinsic
CD300A	9.995729	1.78	Extrinsic
C3AR1	9.9701823	1.88	Extrinsic
TAP2	9.8921552	1.69	Extrinsic
HLA-DRB1	9.8537477	2.41	Extrinsic
PRIM1	9.7894058	1.4	Intrinsic
CCNB1	9.7169622	1.62	Intrinsic
CD86	9.6346643	1.78	Extrinsic

ATP7A	9.6058667	-1.48	Intrinsic
SIGLEC9	9.4338831	1.93	Extrinsic
CD53	9.3170357	2.03	Extrinsic
PCLAF	9.1975509	1.65	Intrinsic
LMNB1	9.1441948	1.42	Intrinsic
CD209	8.7312353	2.15	Extrinsic
ADAMDEC1	8.4634085	1.91	Extrinsic
ERBB2	8.3342401	-1.81	Intrinsic
PSMB9	8.2141712	1.68	Extrinsic
ITGB2	8.2021328	1.84	Extrinsic
CTSW	8.2019558	1.84	Extrinsic
NSD1	8.0970647	-1.3	Intrinsic
TLR4	8.0094864	1.65	Extrinsic
LILRB2	7.9535134	1.91	Extrinsic
KAT6B	7.9463474	-1.43	Intrinsic
CDCA8	7.908568	1.53	Intrinsic
KIF2C	7.8634048	1.64	Intrinsic
SRM	7.8106478	1.4	Intrinsic
GPX1	7.8019435	1.48	Intrinsic
PIK3CB	7.6888667	1.35	Intrinsic
GNLY	7.5283591	2.06	Extrinsic
CCL5	7.3616888	2.72	Extrinsic
GZMA	7.3589661	2.24	Extrinsic
MYBL2	7.3133823	1.95	Intrinsic
RUVBL1	7.2820733	1.45	Intrinsic
IRF1	7.236472	1.85	Extrinsic
MSLN	7.1509685	2.3	Extrinsic
IL10RA	7.1421187	1.75	Extrinsic
CD4	6.9144379	1.68	Extrinsic
BCL2L1	6.7691304	1.39	Extrinsic
UBE2C	6.7544829	1.94	Intrinsic
RBBP5	6.7533955	-1.29	Intrinsic
CD38	6.6936116	1.85	Extrinsic
FASLG	6.6273815	2.19	Intrinsic
IL2RB	6.5646715	1.93	Extrinsic
ARID4B	6.5574569	-1.38	Intrinsic
NCAPG	6.5512608	1.5	Intrinsic
OAS3	6.4690085	1.67	Extrinsic
PIK3R5	6.4114815	1.75	Intrinsic
CAV1	6.3690473	2.07	Intrinsic
MS4A4A	6.2625204	1.99	Extrinsic
CLEC4A	6.0410451	2.01	Extrinsic
KLRD1	6.0374337	2.15	Extrinsic
BAX	6.0337196	1.26	Intrinsic
MYC	6.0084481	1.56	Intrinsic
OAS1	5.98349	1.52	Extrinsic
ITGAM	5.9829251	1.79	Extrinsic
ITGA5	5.9066126	1.56	Intrinsic
TAPBP	5.8615461	1.33	Extrinsic

CCNA2	5.7824406	1.36	Intrinsic
SMO	5.7410309	-2.02	Intrinsic
CD163	5.7249899	2.06	Extrinsic
EOMES	5.6548774	1.64	Extrinsic
KDM6A	5.6475676	-1.45	Intrinsic
GZMK	5.5740863	2.38	Extrinsic
CD244	5.5169676	1.71	Extrinsic
PSMB10	5.5139717	1.46	Extrinsic
CDC20	5.5036848	1.76	Intrinsic
CCR5	5.5019449	1.88	Extrinsic
MCM2	5.4992169	1.51	Intrinsic
PA2G4	5.4101147	1.19	Extrinsic
BUB1B	5.3976924	1.45	Intrinsic
IFNG	5.3699566	2	Extrinsic

B. Adenocarcinoma.

	**ANOVA value**	**Fold change**	**Pathway**
MYC	20.26	2.32	Intrinsic
RRM2	17.29	2.15	Intrinsic
MAPK14	16.59	-1.57	Intrinsic
MYBL2	16.07	2.28	Intrinsic
AXL	16.07	2.32	Intrinsic
CDCA5	14.56	2.14	Intrinsic
AKT1S1	14.43	1.58	Intrinsic
TGFB1	13.79	2.19	Intrinsic
CD209	13.74	2.7	Extrinsic
CDK12	13.73	-1.87	Intrinsic
PCLAF	13.49	2.04	Intrinsic
BAX	13.33	1.51	Intrinsic
CCL13	12.53	4	Extrinsic
LAG3	12.46	2.86	Extrinsic
HMOX1	11.96	2.47	Intrinsic
ARID4A	11.67	-1.64	Intrinsic
SLC1A5	11.49	1.54	Intrinsic
ARID4B	11.30	-1.78	Intrinsic
DNMT1	11.05	1.82	Intrinsic
BCL2L1	10.78	1.88	Extrinsic
MKI67	10.67	2.11	Intrinsic
PGK1	10.61	1.76	Intrinsic
HLA-C	10.56	1.76	Extrinsic
SRM	10.28	1.6	Intrinsic
KMT2A	10.01	-1.52	Intrinsic
TUBG1	9.76	1.5	Intrinsic
HJURP	9.75	1.95	Intrinsic
EIF2AK3	9.61	-1.48	Intrinsic
IL15RA	9.59	1.9	Extrinsic
MSLN	9.06	2.43	Extrinsic
TAP1	8.86	1.88	Extrinsic
E2F1	8.85	1.79	Intrinsic

BRAF	8.83	-1.5	Intrinsic
ATP7A	8.76	-1.69	Intrinsic
CD8A	8.61	3.1	Extrinsic
NKG7	8.41	3.19	Extrinsic
SFN	8.40	2.39	Intrinsic
TAP2	8.39	1.6	Extrinsic
BUB1	8.36	1.81	Intrinsic
MST1R	8.28	1.99	Extrinsic
CCL5	8.20	2.83	Extrinsic
CCL2	8.19	2.35	Extrinsic
TET2	8.13	-1.99	Intrinsic
NSD1	8.08	-1.37	Intrinsic
KAT6B	8.01	-1.42	Intrinsic
FASLG	7.94	1.93	Intrinsic
CAV1	7.81	2.67	Intrinsic
AURKB	7.79	2.2	Intrinsic
GREM1	7.79	3.2	Intrinsic
PIK3R2	7.67	1.74	Intrinsic
CYBB	7.67	2.09	Extrinsic
ABL1	7.64	1.55	Intrinsic
CD68	7.59	2.17	Extrinsic
EIF4EBP1	7.57	1.74	Intrinsic
IL2RA	7.56	2.21	Extrinsic
ATF2	7.55	-1.32	Intrinsic
PMAIP1	7.29	2.05	Intrinsic
CCND1	7.20	1.84	Intrinsic
PIK3R4	7.20	-1.52	Intrinsic
MGA	7.19	-1.61	Intrinsic
AURKA	7.19	1.65	Intrinsic
MCM2	7.16	1.84	Intrinsic
LAMA4	7.11	2.43	Intrinsic
NT5E	7.05	1.86	Extrinsic
KDM6A	7.04	-1.85	Intrinsic
TPI1	6.97	1.47	Intrinsic
EPC1	6.84	-1.94	Intrinsic
TRAF6	6.83	-1.35	Extrinsic
CD274	6.81	1.91	Extrinsic
FANCA	6.79	1.54	Intrinsic
PARP3	6.74	-1.57	Intrinsic
ATG2B	6.60	-1.64	Intrinsic
NFKB2	6.56	1.49	Extrinsic
MAP2K1	6.52	1.43	Intrinsic
PRIM1	6.52	1.43	Intrinsic
DAPP1	6.48	-2.04	Extrinsic
TNS4	6.34	2.71	Intrinsic
DOCK1	6.26	-1.65	Intrinsic
ASH1L	6.24	-1.42	Intrinsic
CD14	6.18	2.18	Extrinsic
CCR1	6.17	1.75	Extrinsic

RUVBL1	6.17	1.7	Intrinsic
GLUD1	6.16	1.61	Intrinsic
ZMYM2	6.09	-1.99	Intrinsic
IL7R	6.06	2.47	Extrinsic
FCGR3A/B	6.03	1.8	Extrinsic
LTBR	6.01	2.01	Extrinsic
ERBB2	5.97	-1.92	Intrinsic
STAT1	5.96	1.56	Extrinsic
CDK4	5.96	1.52	Extrinsic
PDCD1LG2	5.84	1.68	Extrinsic
CCNB1	5.84	1.82	Intrinsic
GABPA	5.78	-1.55	Intrinsic
TPX2	5.56	1.85	Intrinsic
BYSL	5.52	1.44	Intrinsic
TIMELESS	5.51	1.51	Intrinsic
KEAP1	5.46	1.48	Intrinsic
NCOA2	5.45	-1.49	Intrinsic
VEGFB	5.44	-1.42	Intrinsic
IL9R	5.43	2.23	Extrinsic

C. Squamous cell carcinoma.

	**ANOVA value**	**Fold change**	**Pathway**
CCR1	17.49	3.04	Extrinsic
FCER1G	16.13	3.33	Extrinsic
SERPINE1	15.91	2.91	Intrinsic
CD38	15.10	3.46	Extrinsic
GNLY	14.65	4.38	Extrinsic
CYBB	14.33	3.15	Extrinsic
IL15RA	14.05	1.89	Extrinsic
CCL2	14.00	3.73	Extrinsic
VAV1	13.73	2.61	Extrinsic
CD274	13.48	2.19	Extrinsic
GZMB	13.22	4.16	Extrinsic
GZMH	13.20	3.82	Extrinsic
CD300A	12.85	2.7	Extrinsic
HAVCR2	12.65	2.6	Extrinsic
ICAM1	11.85	2.13	Extrinsic
CTSW	11.79	3.38	Extrinsic
MS4A4A	11.67	2.95	Extrinsic
SIGLEC9	10.97	2.73	Extrinsic
PDCD1LG2	10.74	4.19	Extrinsic
CXCL10	10.35	6.8	Extrinsic
CSF2RA	10.26	2.87	Extrinsic
PIK3R5	10.24	2.61	Intrinsic
CD86	10.14	2.46	Extrinsic
LYN	10.13	1.76	Extrinsic
PDCD1	9.69	2.52	Extrinsic
PTPRC	9.68	2.63	Extrinsic

LY96	9.65	2.45	Extrinsic
CD53	9.59	2.59	Extrinsic
PRF1	9.53	2.95	Extrinsic
PIK3CB	9.44	1.67	Intrinsic
NKG7	9.23	3.44	Extrinsic
ITGB2	9.20	2.57	Extrinsic
CMKLR1	8.95	2.65	Extrinsic
ACKR2	8.90	3.26	Extrinsic
ITGAM	8.30	2.26	Extrinsic
RPA3	8.22	1.38	Intrinsic
KIF2C	7.91	1.71	Intrinsic
ABCG2	7.83	2.28	Intrinsic
UBE2C	7.81	1.68	Intrinsic
FCGR3A/B	7.76	2.81	Extrinsic
RAF1	7.75	1.36	Intrinsic
LILRB2	7.65	2.78	Extrinsic
CCR2	7.62	2.86	Extrinsic
NFATC2	7.42	2.38	Extrinsic
TRAT1	7.40	2.56	Extrinsic
NCR1	7.33	2.38	Extrinsic
IL10RA	7.21	2.34	Extrinsic
NCF2	7.19	2.62	Intrinsic
CGAS	7.11	1.89	Extrinsic
C3AR1	7.00	2.16	Extrinsic
LAG3	6.99	3.09	Extrinsic
CD3E	6.93	2.52	Extrinsic
IRF1	6.93	2.31	Extrinsic
ADAMDEC1	6.82	2.18	Extrinsic
KDM1A	6.81	1.49	Intrinsic
TERT	6.71	2.82	Intrinsic
HLA-DQA1	6.67	5.54	Extrinsic
CDCA8	6.60	1.73	Intrinsic
KIR2DL4	6.52	2.01	Extrinsic
B2M	6.47	2.12	Extrinsic
JAG1	6.38	-3.03	Intrinsic
CD244	6.31	2.66	Extrinsic
CD14	6.25	2.18	Extrinsic
LAPTM5	6.24	2.25	Extrinsic
CD4	6.20	2.11	Extrinsic
ELOVL5	6.13	1.84	Intrinsic
CDC25A	6.13	1.59	Intrinsic
IQGAP1	6.07	1.48	Intrinsic
CCNA2	6.03	1.45	Intrinsic
GREM1	5.99	2.87	Intrinsic
RAD51	5.93	1.66	Intrinsic
AMOTL2	5.67	1.66	Intrinsic
ADORA2A	5.66	1.86	Extrinsic
CD27	5.65	2.96	Extrinsic
ATOX1	5.52	1.62	Intrinsic

BCL2A1	5.44	3.72	Extrinsic
TAP1	5.43	2.27	Extrinsic
TBX21	5.39	2.01	Extrinsic
GZMA	5.39	2.73	Extrinsic
STAT1	5.34	2.06	Extrinsic
HSD11B1	5.21	3.06	Intrinsic
CCR5	5.19	2.88	Extrinsic
CD247	5.15	2.14	Extrinsic
AURKA	5.14	1.58	Intrinsic
NF1	5.12	-1.53	Intrinsic
MNDA	5.05	2.7	Extrinsic
SMO	4.97	-3.09	Intrinsic
CD68	4.95	2.08	Extrinsic
FLT1	4.93	2	Intrinsic
LMNB1	4.92	1.47	Intrinsic
PTCH1	4.89	-2.16	Intrinsic
FZD7	4.88	-2.99	Intrinsic
KLRD1	4.84	2.72	Extrinsic
EOMES	4.67	2.31	Extrinsic
PTGER2	4.63	1.79	Extrinsic
ARID1B	4.63	-1.31	Intrinsic
SH3PXD2A	4.58	-1.58	Intrinsic
EPB41L3	4.55	1.67	Intrinsic
GPX1	4.51	1.52	Intrinsic
SIGLEC5	4.49	2.04	Extrinsic

Table S4. The determination of the optimal number of genes.

Intrinsic pathway related genes in total 57 NSCLC.

	kNN	SVM	RF	NN	NB	LR	AUC_sum
5 features	0.730	0.607	0.736	0.808	0.778	0.825	4.484
10 features	0.691	0.722	0.709	0.792	0.759	0.699	4.372
15 features	0.741	0.830	0.673	0.766	0.802	0.787	4.599
20 features	0.736	0.805	0.742	0.804	0.830	0.699	4.616
25 features	0.742	0.851	0.599	0.815	0.851	0.747	4.605
30 features	0.743	0.879	0.828	0.808	0.858	0.719	4.835

Extrinsic pathway related genes in total 57 NSCLC.

	kNN	SVM	RF	NN	NB	LR	AUC_sum
5 features	0.745	0.780	0.807	0.816	0.823	0.785	4.756
10 features	0.726	0.834	0.900	0.801	0.823	0.344	4.428
15 features	0.813	0.825	0.779	0.806	0.839	0.325	4.387
20 features	0.705	0.827	0.783	0.769	0.829	0.271	4.184
25 features	0.582	0.827	0.766	0.757	0.820	0.456	4.208
30 features	0.520	0.815	0.722	0.781	0.820	0.505	4.163

Intrinsic pathway related genes in lung adenocarcinoma.

	kNN	SVM	RF	NN	NB	LR	AUC_sum
5 features	0.857	0.964	0.938	0.952	0.964	0.946	5.621

10 features	0.884	0.988	0.887	0.982	0.982	0.976	5.699
15 features	0.902	0.976	0.929	0.946	0.994	1	5.747
20 features	0.857	1	0.821	1	1	0.982	5.66
25 features	0.857	1	0.765	1	0.994	0.946	5.562
30 features	0.839	1	0.893	1	0.994	0.929	5.655

Extrinsic pathway related genes in lung adenocarcinoma.

	kNN	SVM	RF	NN	NB	LR	AUC_sum
5 features	0.726	0.851	0.744	0.756	0.851	0.702	4.63
10 features	0.622	0.905	0.747	0.732	0.851	0.795	4.652
15 features	0.696	1	0.884	0.881	0.839	0.673	4.973
20 features	0.783	1	0.696	0.893	0.887	0.655	4.914
25 features	0.812	0.994	0.738	0.839	0.899	0.5	4.782
30 features	0.827	0.994	0.804	0.923	0.923	0.268	4.739

Intrinsic pathway related genes in lung squamous cell carcinoma.

	kNN	SVM	RF	NN	NB	LR	AUC_sum
5 features	0.708	0.808	0.783	0.842	0.900	0.742	4.783
10 features	0.638	0.792	0.742	0.758	0.867	0.383	4.18
15 features	0.708	0.808	0.721	0.717	0.858	0.658	4.47
20 features	0.671	0.842	0.646	0.775	0.833	0.717	4.484
25 features	0.671	0.833	0.754	0.775	0.825	0.742	4.6
30 features	0.671	0.825	0.754	0.775	0.850	0.733	4.608

Extrinsic pathway related genes in lung squamous cell carcinoma.

	kNN	SVM	RF	NN	NB	LR	AUC_sum
5 features	0.783	0.6	0.908	0.692	0.842	0.458	4.283
10 features	0.675	0.792	0.871	0.675	0.883	0.525	4.421
15 features	0.675	0.633	0.842	0.608	0.867	0.212	3.837
20 features	0.817	0.750	0.850	0.658	0.892	0.275	4.242
25 features	0.817	0.692	0.867	0.750	0.883	0.354	4.363
30 features	0.75	0.708	0.775	0.667	0.867	0.342	4.109

Table S5. Classification performances of machine-learning models.

A. 30 intrinsic pathway-associated genes in non-small cell carcinoma.

Method	AUC	Accuracy	F1	Precision	Recall	Sum
K-nearest neighbors	0.743	0.807	0.779	0.788	0.807	3.924
Logistic regression	0.719	0.807	0.809	0.812	0.807	3.954
Naïve Bayes	0.858	0.772	0.790	0.863	0.772	4.055
Neural network	0.808	0.860	0.855	0.854	0.860	4.237
Random forest	0.828	0.825	0.804	0.811	0.825	4.093
Support vector machine	0.879	0.825	0.793	0.821	0.825	4.143

B. 5 extrinsic pathway-associated genes in non-small cell carcinoma.

Method	AUC	Accuracy	F1	Precision	Recall	Sum
K-nearest neighbors	0.745	0.719	0.669	0.639	0.719	3.491
Logistic regression	0.785	0.860	0.855	0.854	0.860	4.214

Naïve Bayes	0.823	0.754	0.771	0.816	0.754	3.918
Neural network	0.816	0.860	0.855	0.854	0.860	4.245
Random forest	0.807	0.807	0.798	0.794	0.807	4.013
Support vector machine	0.780	0.860	0.834	0.881	0.860	4.215

C. 15 intrinsic pathway-associated genes in adenocarcinoma.

Method	AUC	Accuracy	F1	Precision	Recall	Sum
K-nearest neighbors	0.902	0.871	0.85	0.889	0.871	4.383
Logistic regression	1	0.968	0.967	0.969	0.968	4.872
Naïve Bayes	0.994	0.871	0.879	0.918	0.871	4.533
Neural network	0.946	0.903	0.9	0.9	0.903	4.552
Random forest	0.929	0.903	0.893	0.914	0.903	4.542
Support vector machine	0.976	0.935	0.931	0.940	0.935	4.717

D. 15 extrinsic pathway-associated genes in adenocarcinoma.

Method	AUC	Accuracy	F1	Precision	Recall	Sum
K-nearest neighbors	0.696	0.774	0.676	0.599	0.774	3.519
Logistic regression	0.673	0.806	0.775	0.786	0.806	3.846
Naïve Bayes	0.839	0.677	0.704	0.816	0.677	3.713
Neural network	0.881	0.935	0.931	0.940	0.935	4.622
Random forest	0.884	0.806	0.775	0.786	0.806	4.057
Support vector machine	1	0.935	0.931	0.94	0.935	4.741

E. 5 intrinsic pathway-associated genes in squamous cell carcinoma.

Method	AUC	Accuracy	F1	Precision	Recall	Sum
K-nearest neighbors	0.708	0.808	0.750	0.846	0.808	3.92
Logistic regression	0.742	0.769	0.780	0.799	0.799	3.889
Naïve Bayes	0.900	0.846	0.853	0.871	0.846	4.316
Neural network	0.842	0.923	0.917	0.930	0.923	4.535
Random forest	0.783	0.808	0.782	0.789	0.808	3.97
Support vector machine	0.808	0.846	0.834	0.837	0.846	4.171

F.10 extrinsic pathway-associated genes in squamous cell carcinoma.

Method	AUC	Accuracy	F1	Precision	Recall	Sum
K-nearest neighbors	0.675	0.769	0.722	0.724	0.769	3.659
Logistic regression	0.525	0.654	0.674	0.710	0.654	3.217
Naïve Bayes	0.883	0.731	0.751	0.823	0.731	3.919
Neural network	0.675	0.731	0.722	0.715	0.731	3.574
Random forest	0.871	0.808	0.801	0.798	0.808	4.086
Support vector machine	0.792	0.846	0.834	0.837	0.846	4.155

Table S6. Classification performances of machine-learning models for ayers 13 genes signature.

A. Adenocarcinoma.

Method	AUC	Accuracy	F1	Precision	Recall	Sum
K-nearest neighbors	0.598	0.742	0.659	0.594	0.742	3.335
Logistic regression	0.571	0.742	0.742	0.742	0.742	3.539
Naïve Bayes	0.714	0.581	0.614	0.742	0.581	3.232
Neural network	0.452	0.645	0.607	0.573	0.645	2.922
Random forest	0.506	0.710	0.643	0.587	0.710	3.156
Support vector machine	0.137	0.742	0.659	0.594	0.742	2.874

B. Squamous cell carcinoma.

Method	AUC	Accuracy	F1	Precision	Recall	Sum
K-nearest neighbors	0.517	0.731	0.650	0.585	0.731	3.214
Logistic regression	0.850	0.923	0.923	0.923	0.923	4.542
Naïve Bayes	0.783	0.692	0.715	0.868	0.692	3.75
Neural network	0.742	0.731	0.722	0.715	0.731	3.641
Random forest	0.733	0.769	0.752	0.745	0.769	3.768
Support vector machine	0.500	0.769	0.669	0.592	0.769	3.299

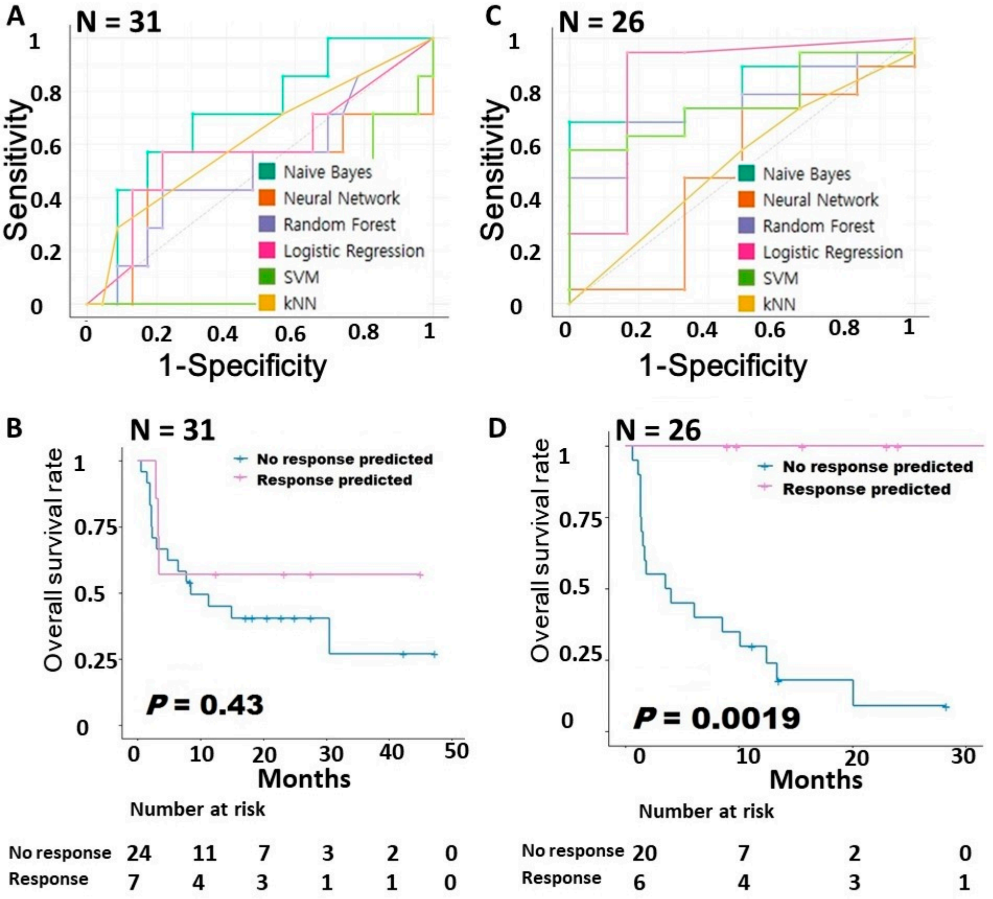

Figure S1. Building a predictive model for responsiveness to PD-1 inhibitors using Ayers' 13 extended immune gene signatures. (**A**) Comparison of the AUCs for Ayers' 13 extended immune gene signatures in LUAD according to six machine learning models. (**B**) Survival analysis according to Ayers' 13 ex-tended immune gene signatures in LUAD. (**C**) Comparison of the AUCs for Ayers' 13 extended immune gene signatures in LUSC according to six machine learning models. (**D**) Survival analysis according to Ayers' 13 extended immune gene signatures in LUSC. AUC, area under the

ROC curve; LUAD, lung adenocarcinoma; LUSC, lung squamous cell carcinoma; PD-1, programmed cell death protein 1.

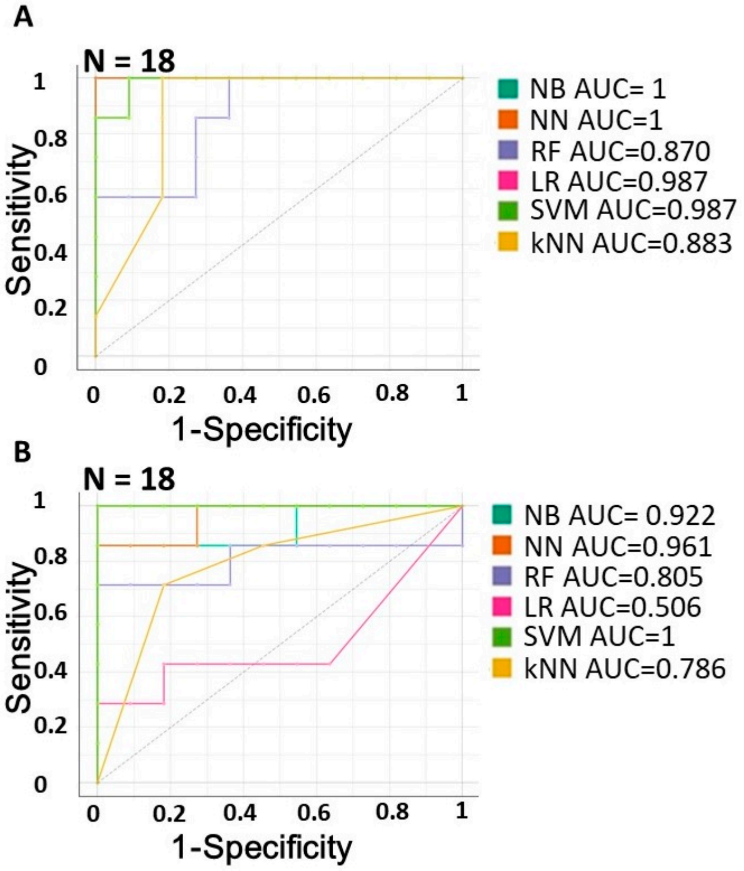

Figure S2. Building a predictive model for responsiveness to PD-1 inhibitors according to EGFR mutation. (**A**) Comparison of the AUCs for 15 intrinsic pathway–associated genes in LUAD in EGFR negative group. (**B**) Comparison of the AUCs for 15 extrinsic pathway–associated genes in LUAD in EGFR negative group.

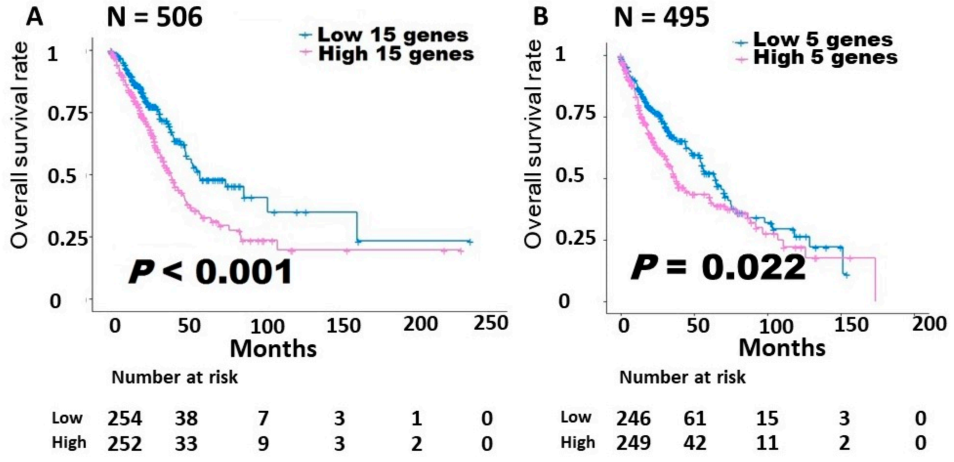

Figure S3. Survival analysis according to the intrinsic gene signature in TCGA data. (**A**) Overall survival according to the intrinsic gene signature in TCGA data for LUAD. (**B**) Overall survival according to the intrinsic gene signature in TCGA data for LUSC. LUAD, lung adenocarcinoma; LUSC, lung squamous cell carcinoma; TCGA, The Cancer Genome Atlas.

Data S1.

The nCounter® Tumor Signaling 360 contains 760 genes and up to 20 genes can be added. Currently, the most widely used method for predicting response to PD-1 inhibitors is PD-L1 immunohistochemistry[1]. Therefore, we judged that the intrinsic gene for predicting the response to PD-1 inhibitor would also be closely related to PD-L1 expression. We performed gene set enrichment analysis (GSEA) on RNA-sequencing data from cBioportal to confirm the intrinsic genes associated with PD-L1 expression[2]. Two datasets (TCGA, Firehose Legacy and OncoSG) were used for lung adenocarcinoma, and one dataset was used for lung squamous cell carcinoma (TCGA, Firehose Legacy). In GSEA analysis, if the p value was less than 0.05 and the False Discovery Rate (FDR) was less than 0.25, the pathways were considered statistically significant. In two lung adenocarcinoma dataset, seven intrinsic pathways (KRAS_SIGNALING_UP, APOPTOSIS, HYPOXIA, EPITHELIAL_MESENCHYMAL_ TRANSITION, PI3K_AKT_MTOR_SIGNALING, APICAL_SURFACE, APICAL_ JUNCTION) increased expression in the PD-L1 expression upregulated group. In lung squamous cell carcinoma dataset, six intrinsic pathways (HEME_METABOLISM, REACTIVE_OXYGEN_SPECIES_PATHWAY, KRAS_SIGNALING_UP, APICAL_ SURFACE, XENOBIOTIC_ METABOLISM, PI3K_AKT_MTOR_SIGNALING) increased expression in the PD-L1 expression upregulated group. Each pathway contains a gene list. In lung adenocarcinoma, genes with a high rank were selected while overlapping in the TCGA and OncoSG datasets in the entire pathway. Similarly, in lung squamous cell carcinoma, a gene with a high rank in the entire pathway was selected. The finally selected genes were not included in the nCounter® Tumor Signaling 360 Panel, but 20 overlapping genes were selected from the lung adenocarcinoma and lung squamous cell carcinoma datasets. The added genes are FCER1G, LAPTM5, IKZF1, TNFRSF1B, RALB, DAPP1, IRF8, CLEC4A, EPB41L3, ADAMDEC1, PRDM1, GFPT2, CD37, ACTR3, MAP4K1, F13A1, NRP1, HBEGF, MKNK1, DCBLD2.

1. Shukuya T, Carbone DP. Predictive Markers for the Efficacy of Anti-PD-1/PD-L1 Antibodies in Lung Cancer. *J Thorac Oncol*. 2016;11:976-88.
2. Cerami E, Gao J, Dogrusoz U, et al. The cBio cancer genomics portal: an open platform for exploring multidimensional cancer genomics data. *Cancer Discov*. 2012;2:401-4.

Data S2.

Ayers et al 18 expanded immune gene signature: CD3D, IL2RG, IDO1, NKG7, CIITA, HLA-E, CD3E, CXCR6, CCL5, LAG3, GZMK, TAGAP, CD2, CXCL10, HLA-DRA, STAT1, CXCL13, GZMB.

Ayers et al 13 expanded immune gene signature in our study: CD3D, IL2RG, IDO1, NKG7, HLA-E, CD3E, CXCR6, CCL5, LAG3, GZMK, CXCL10, STAT1, GZMB.
